# MR CLEAN-LATE, a multicenter randomized clinical trial of endovascular treatment of acute ischemic stroke in The Netherlands for late arrivals: study protocol for a randomized controlled trial

**DOI:** 10.1186/s13063-021-05092-0

**Published:** 2021-02-24

**Authors:** F. A. V. ( Anne) Pirson, Wouter H. Hinsenveld, Robert-Jan B. Goldhoorn, Julie Staals, Inger R. de Ridder, Wim H. van Zwam, Marianne A. A. van Walderveen, Geert J. Lycklama à Nijeholt, Maarten Uyttenboogaart, Wouter J. Schonewille, Aad van der Lugt, Diederik W. J. Dippel, Yvo B. W. E. M. Roos, Charles B. L. M. Majoie, Robert J. van Oostenbrugge, on behalf of the MR CLEAN-LATE investigators

**Affiliations:** 1 Department of Neurology, Maastricht University Medical Center, Postbus 5800, Maastricht, 6202 AZ The Netherlands; 2 Department of Radiology, Maastricht University Medical Center, Maastricht, The Netherlands; 3 Department of Radiology, Leiden University Medical Center, Leiden, The Netherlands; 4 Department of Radiology, Haaglanden Medical Center, The Hague, The Netherlands; 5 Department of Neurology and Department of Radiology, University of Groningen, Groningen, The Netherlands; 6 Department of Neurology, Sint Antonius Hospital, Nieuwegein, The Netherlands; 7 Department of Radiology and Nuclear Medicine, Erasmus MC University Medical Center, Rotterdam, The Netherlands; 8 Department of Neurology, Erasmus MC University Medical Center, Rotterdam, The Netherlands; 9 Department of Neurology, Amsterdam University Medical Center, location AMC, Amsterdam, The Netherlands; 10 Department of Radiology and Nuclear Medicine, Amsterdam University Medical Center, location AMC, Amsterdam, The Netherlands

**Keywords:** Endovascular treatment, Thrombectomy, Acute ischemic stroke, Randomized controlled trial, Late arrivals

## Abstract

**Background:**

Endovascular therapy (EVT) for acute ischemic stroke due to proximal occlusion of the anterior intracranial circulation, started within 6 h from symptom onset, has been proven safe and effective. Recently, EVT has been proven effective beyond the 6-h time window in a highly selected population using CT perfusion or MR diffusion. Unfortunately, these imaging modalities are not available in every hospital, and strict selection criteria might exclude patients who could still benefit from EVT. The presence of collaterals on CT angiography (CTA) may offer a more pragmatic imaging criterion that predicts possible benefit from EVT beyond 6 h from time last known well. The aim of this study is to assess the safety and efficacy of EVT for patients treated between 6 and 24 h from time last known well after selection based on the presence of collateral flow.

**Methods:**

The MR CLEAN-LATE trial is a multicenter, randomized, open-label, blinded endpoint trial, aiming to enroll 500 patients. We will investigate the efficacy of EVT between 6 and 24 h from time last known well in acute ischemic stroke due to a proximal intracranial anterior circulation occlusion confirmed by CTA or MRA. Patients with any collateral flow (poor, moderate, or good collaterals) on CTA will be included. The inclusion of poor collateral status will be restricted to a maximum of 100 patients. In line with the current Dutch guidelines, patients who fulfill the characteristics of included patients in DAWN and DEFUSE 3 will be excluded as they are eligible for EVT as standard care. The primary endpoint is functional outcome at 90 days, assessed with the modified Rankin Scale (mRS) score. Treatment effect will be estimated with ordinal logistic regression (shift analysis) on the mRS at 90 days. Secondary endpoints include clinical stroke severity at 24 h and 5–7 days assessed by the NIHSS, symptomatic intracranial hemorrhage, recanalization at 24 h, follow-up infarct size, and mortality at 90 days,

**Discussion:**

This study will provide insight into whether EVT is safe and effective for patients treated between 6 and 24 h from time last known well after selection based on the presence of collateral flow on CTA.

**Trial registration:**

NL58246.078.17, ISRCTN19922220, Registered on 11 December 2017

**Supplementary Information:**

The online version contains supplementary material available at 10.1186/s13063-021-05092-0.

## Background

Since the publication in 2015 of several trials showing benefit from endovascular therapy (EVT), it has become the new standard for treatment of acute ischemic stroke (AIS) due to large vessel anterior circulation occlusion within 6 h [1–5]. A subsequent pooled meta-analysis of individual patient data from these trials showed that EVT started within 6 h of symptom onset leads to an additional 20% of patients being functionally independent at 90 days after treatment [6]. The same analysis also showed that the association between thrombectomy and improved outcomes was significant up to 7.3 h after symptom onset. Of the original trials, REVASCAT and ESCAPE included patients in the 6–8-h and 6–12-h time window, respectively, yet patient numbers in these time intervals were too small to draw firm conclusions on the efficacy of EVT [2, 3]. More recently, the trials DAWN and DEFUSE 3 showed significant benefit of EVT for patients with a distal intracranial carotid artery occlusion/ACM-M1 occlusion beyond 6 h from time last known well for a highly selected patient group with severe neurological deficit and small infarct core volumes assessed by CT perfusion or MR diffusion/perfusion imaging [7, 8]. However, due to selection, the generalizability is low, and practical implementation of these results might be limited as many primary stroke centers do not routinely perform perfusion imaging. Additionally, the benefit of EVT in patients not meeting the clinical and imaging characteristics of these trials is unknown. Based on the results from DAWN and DEFUSE 3, in which EVT was shown highly effective, the benefit of EVT for a much larger patient group in the 6- to 24-h time window could be expected. Ischemic core volumes and perfusion mismatch seem to be associated with collateral circulation grades on CT angiography (CTA) [9–11]. The ESCAPE trial suggested that the inclusion of patients with only moderate to good collateral grades had led to a larger effect size in their study compared with the other original trials that did not use advanced imaging selection criteria. Moreover, a post hoc analysis of MR CLEAN showed that collateral status on CTA was associated with EVT treatment effect: patients with higher collateral grades showed larger treatment effects [12]. Therefore, the presence of collaterals could be an adequate and practical alternative for selecting patients with a potential treatment benefit beyond the 6-h time window.

### Research question

**Fig. 1:**
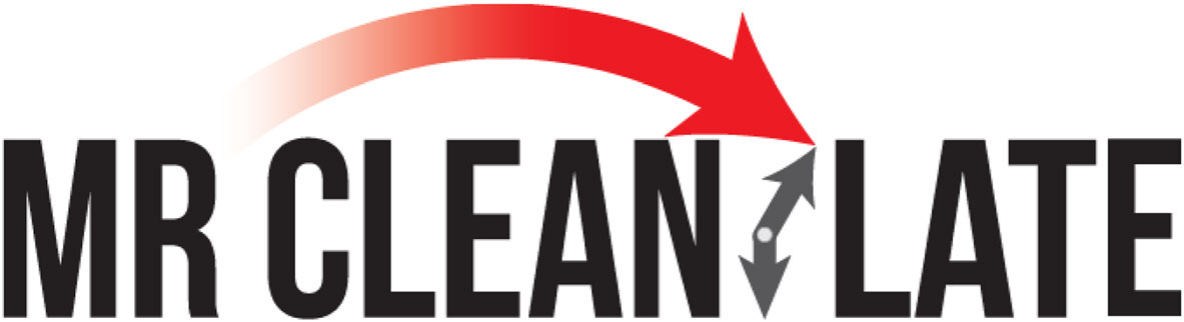
Trial logo

The primary objective of MR CLEAN-LATE (Fig. 1) is to assess the safety and efficacy of EVT in addition to best medical treatment compared with best medical treatment alone on functional outcome in patients with AIS, caused by an intracranial large vessel occlusion of the anterior circulation and any poor, moderate, or good collaterals confirmed by neuro-imaging presenting between 6 and 24 h from time last known well.

Secondary objectives are to assess the effect of EVT on the early neurological outcome, assessed with the National Institutes of Health Stroke Scale (NIHSS) score, the occurrence of symptomatic intracranial hemorrhage (sICH), mortality at 90 days, recanalization on CTA or MRA, and infarct size on MRI or on non-contrast CT (NCCT).

**Fig. 2:**
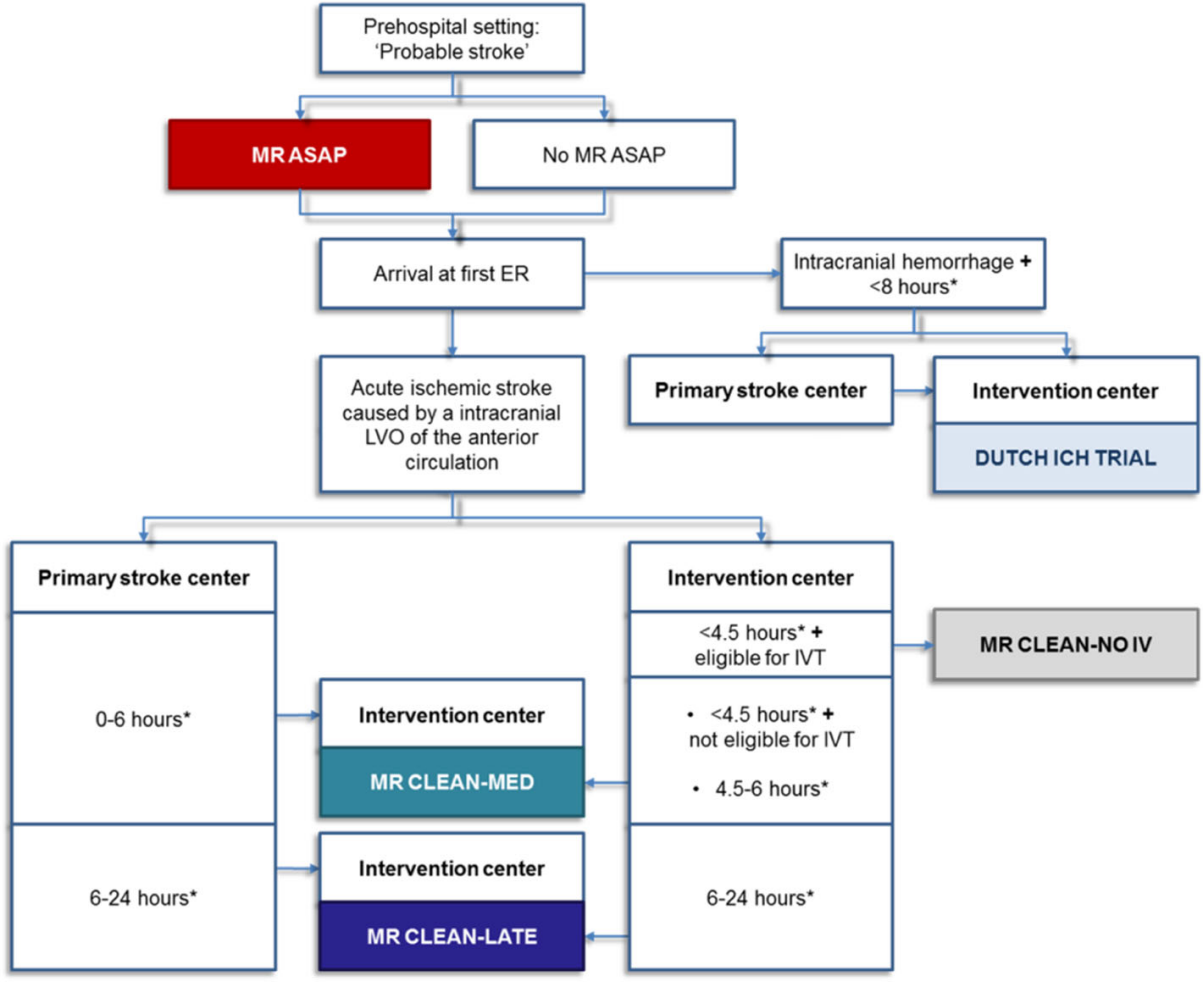
Flow of patients in the CONTRAST consortium. MR ASAP, Multicentre Randomised trial of Acute Stroke treatment in the Ambulance with a nitroglycerin Patch; ER, emergency room; DUTCH ICH pilot, a prospective, multicenter, randomized open, blinded end-point clinical trial of minimally invasive surgery, steroids or both in patients with spontaneous, non-traumatic supratentorial ICH in The Netherlands; MR CLEAN-MED,: Multicenter Randomized CLinical trial of Endovascular treatment for Acute ischemic stroke in the Netherlands. The effect of periprocedural MEDication: antiplatelet agents, heparin, both or neither; MR CLEAN-NO IV, Intravenous treatment followed by intra-arterial treatment vs direct intra-arterial treatment for acute ischemic stroke caused by a proximal intracranial occlusion; IVT, intravenous thrombolysis with alteplase; MR CLEAN-LATE, Multicenter Randomized Clinical Trial of Endovascular Stroke treatment in The Netherlands for Late arrivals. Considerations: (1) The CONTRAST studies are independent RCT’s. Patients who have been included in MR ASAP may also be included in one of the intervention trials for ischemic or for hemorrhagic stroke. Being eligible for two trials at the same time raises questions whether the trials influence each other’s results. Therefore, we will perform pre-specified subgroup analyses to test for the interaction between the different performed treatments. Further, part of the potential treatment effect in MR ASAP will be represented in the baseline characteristics measured at inclusion in the second trial, such as collaterals, blood pressure, and NIHSS, which we will adjust for in all analyses. (2) At the first ER (either a primary stroke center or a participating intervention center), all patients with a probable diagnosis of acute stroke will undergo non-contrast CT to differentiate between acute cerebral infarction or acute intracranial hemorrhage. When the first ER is a primary stroke center and the patient could be eligible for the DUTCH ICH TRIAL, MR CLEAN-MED, or MR CLEAN-LATE study, the patient should be transferred to a participating intervention center (where inclusion in one of these studies, randomization, and treatment takes place). (3) Patients arriving first at a primary stroke center will generally not be eligible for the MR CLEAN-NO IV, since intravenous thrombolysis with alteplase (IVT) cannot be withheld until after patient transfer to the participating intervention center, unless the perceived contraindications for alteplase are not present anymore upon arrival at the intervention center. Then, inclusion in MR CLEAN-NO IV will have priority over inclusion in other trials. Patients who are eligible for inclusion in MR CLEAN-NO IV (primary presentation at intervention center, < 4.5 h + eligible for IVT) will not be included in MR CLEAN-MED. Patients presenting at the primary stroke center within 6 h (both eligible or not eligible for IVT) could be eligible for the MR CLEAN-MED. Importantly by this scheme, competition between the intervention trials will not occur.

Tertiary objectives are (1) to collect waste biomaterials (retrieved thrombus, aspirated blood) to analyze biochemical and biomechanical properties and their potential for treatment effect modification; (2) to collect and analyze data regarding the deferred consent procedure and its association with patient recall and satisfaction at three months from randomization; (3) to collect and provide data, together with other CONTRAST (Consortium for New Treatments of Acute Stroke: Fig. 2) trials, for studies assessing the efficiency of national EVT implementation, given the availability of EVT hospitals and capacity, and travel times of ambulance services; and (4) to perform a cost-effectiveness analysis on patients receiving EVT between 6 and 24 h from time last known well.

## Methods/design

### Design

**Fig. 3:**
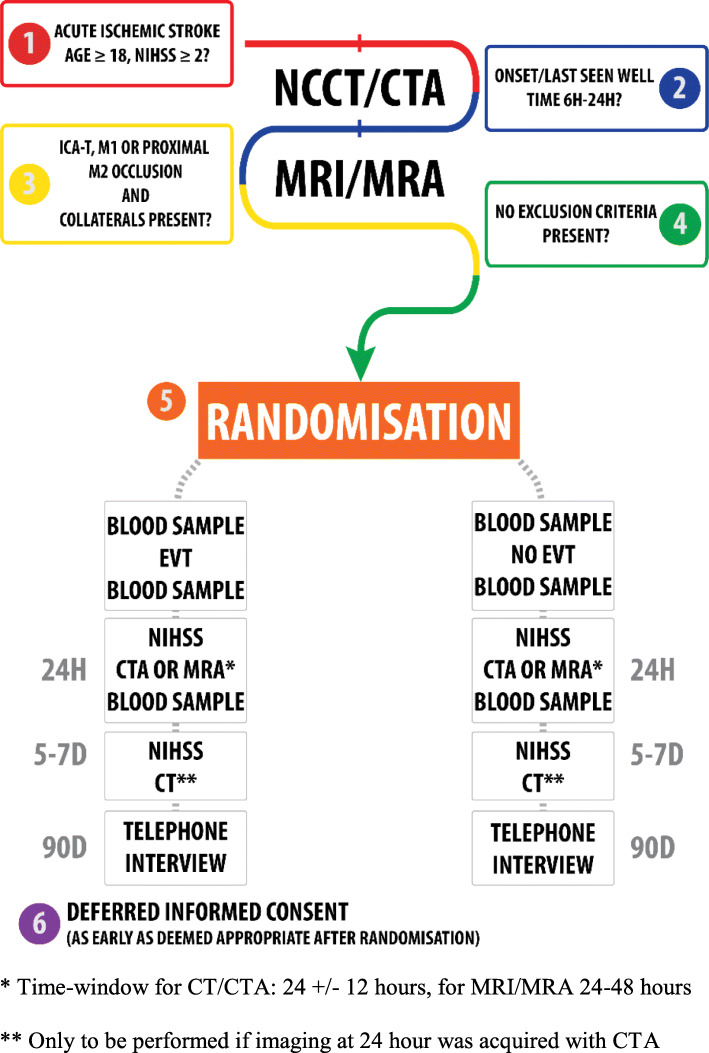
*Time-window for CT/CTA: 24 +/- 12 h, for MRI/MRA 24-48 h. **Only to be performed if imaging at 24 h was acquired with CTA

MR CLEAN-LATE is a multicenter phase III clinical trial with a prospective, randomized treatment allocation, open-label treatment, and blinded endpoint evaluation (PROBE design). The contrast is EVT vs no EVT. The treatment is provided in addition to the best medical treatment. An overview of the main procedures that subjects will undergo is provided in Fig. 3. Patient inclusion started in January 2018.

### Study population

The study population will be drawn from patients presenting at intervention centers within the 6–24 h from time last known well, including patients referred from primary stroke centers. Only subjects who meet the clinical and radiological inclusion criteria are eligible for inclusion in the trial: (1) age ≥ 18; (2) proximal intracranial anterior circulation occlusion (distal intracranial carotid artery or middle (M1/M2)) cerebral artery confirmed by neuro-imaging (CTA or MRA); (3) presence of poor, moderate, or good collateral flow as shown by CTA; (4) non-contrast CT or MRI ruling out intracranial hemorrhage; (5) start of EVT (groin puncture) possible between 6 and 24 h after symptom onset or last seen well, including wake-up strokes; and (6) a score of at least 2 on the NIHSS (Table 1). The exclusion criteria for enrollment in the trial are as follows: (1) pre-stroke disability which interferes with the assessment of functional outcome at 90 days, i.e., mRS > 2; (2) cerebral infarction in the previous 6 weeks with residual neurological deficit or signs of large recent infarction on neuroimaging in the territory of the middle cerebral artery; (3) clinical evidence of hemorrhagic diathesis, confirmed by an INR > 3 and/or a platelet count < 40 × 109/L and/or an APTT > 50 s; and (4) clearly demarcated hypodensity on non-contrast CT in > 1/3 of the middle cerebral artery territory, consistent with current symptoms. Additionally, as per Dutch guidelines, patients with a distal intracranial carotid artery occlusion or ACM-M1 occlusion and NIHSS ≥ 10, an infarct core ≤ 25 ml (75th percentile of included patients in DEFUSE-3), and total ischemic volume/infarct core ratio ≥ 2 on CT perfusion or MR diffusion/perfusion will not be included but receive EVT as standard care (Nederlandse Vereniging voor Neurologie, Herseninfarct en Hersenbloeding, 2020; https://richtlijnendatabase.nl/richtlijn/herseninfarct_en_hersenbloeding/reperfusietherapie_voor_acute_herseninfarct/endovasculaire_trombectomie_evt_bij_herseninfarct.html).

**Table 1:** Inclusion and exclusion criteria

Inclusion criteria	
1. Clinical diagnosis of acute ischemic stroke	
2. Age ≥ 18	
3. Proximal intracranial anterior circulation occlusion (distal intracranial carotid artery or middle (M1/M2)) cerebral artery confirmed by neuro-imaging (CTA or MRA)	
4. Presence of poor, moderate, or good collateral flow as shown by CTA	
5. Non-contrast CT or MRI ruling out intracranial hemorrhage	
6. Start of EVT (groin puncture) possible between 6 and 24 h after symptom onset or last seen well between 6 and 24 h including wake-up strokes	
7. A score of at least 2 on the NIHSS	
8. Written informed consent (deferred)	
Exclusion criteria	
1. Pre-stroke disability which interferes with the assessment of functional outcome at 90 days, i.e., mRS > 2	
2. Cerebral infarction in the previous 6 weeks with residual neurological deficit or signs of large recent infarction on neuroimaging in the territory of the middle cerebral artery	
3. Clinical evidence of hemorrhagic diathesis, confirmed by an INR > 3 and/or a platelet count < 40 × 10^9^/L and/or an APTT > 50 s	
4. Clearly demarcated hypodensity on non-contrast CT in > 1/3 of the middle cerebral artery territory, consistent with current symptoms	
5. Distal intracranial carotid artery occlusion/M1 occlusion, NIHSS ≥ 10, and infarct core ≤ 25 ml and total ischemic volume/infarct core ratio ≥ 2 on CT perfusion or MR diffusion/perfusion	
6. Participation in other trials except for MR ASAP and ARTEMIS	

*CTA* computed tomography angiography, *MRA* magnetic resonance angiography, *NCCT* non-contrast computed tomography, *MRI* magnetic resonance imaging, *EVT* endovascular treatment, *NIH* National Institutes of Health, *mRS* modified Rankin Scale, *INR* international normalized ratio, *APTT* activated partial thromboplastin time

The study is expected to run for 4 years (or until 500 patients are included) in eligible stroke intervention centers in The Netherlands and will be carried out by researchers of the Consortium for New Treatments of Acute Stroke (CONTRAST: Additional file 1: Appendix 1). Initially, all patients with poor collaterals (collaterals filling ≤ 50% of the occluded territory), moderate collaterals (collaterals filing > 50%, but < 100% of the occluded territory), or good collaterals (collaterals filling 100% of the occluded territory) on CTA (single phase) will be included. When a total number of 100 patients with poor collateral status have been reached, further inclusion and randomization of patients with poor collaterals will stop.

### Eligibility criteria for participating centers

Intervention centers will be able to participate if they (1) have experience in conducting acute stroke trials, (2) meet the Dutch quality criteria of the “Nederlandse Vereniging voor Radiologie” and “Nederlandse Vereniging voor Neurologie” to perform EVT (e.g., available for treatment 24 h, 7 days a week; at least 50 EVT procedures performed per year; at least 3 interventionists), (3) have a stroke team (including interventionists) with sufficient experience performing EVT (> 20 EVT procedures performed per physician per year), (4) use one or more devices approved by the trial steering committee (Table 2), and (5) have sufficient experience with the particular device [13].

**Table 2:** List of currently approved treatment devices

Device name	Manufacturer	Description
Solitaire	Medtronic/Covidien	Retrievable stent
Trevo	Stryker	Retrievable stent
Revive	Cerenovus	Retrievable stent
Catch	Balt	Retrievable stent
Embotrap	Cerenovus	Retrievable stent
Eric	Microvention	Retrievable stent
PreSet	Phenox	Retrievable stent
3D Separator	Penumbra	Retrievable stent
Penumbra system	Penumbra	Aspiration catheter system
Sofia	Microvention	Aspiration catheter
Catalyst	Stryker	Aspiration catheter
Syphontrak	InNeuroCo	Aspiration catheter

New devices may be used when they are CE-marked or FDA-approved and after approval by the study executive committee

### Randomization and blinding

The randomization procedure will be computer- and web-based, using permuted blocks. Randomization will take place at the intervention center and will be stratified for the center and for the treatment allocation in an ongoing prehospital randomized intervention trial (MR ASAP; ISRCTN99503308) [14]. Only the treating physician, or local study investigator of the intervention center that is trained for the assessment of trial eligibility, is allowed to enroll participants. The allocation sequence has been generated by the independent trial statistician. Both patient and treating physician will be aware of the treatment assignment. It will not be possible to view the treatment allocation before the patient is registered in the study database, nor will it be possible to remove the patient from the study database. For each patient that withdraws before the final outcome assessment, an additional patient will be included. The primary outcome will be assessed using standardized forms and procedures in a standardized phone interview by a research nurse blinded to treatment allocation [15, 16]. To guarantee unawareness of the research personnel assessing the outcome at 3 months, they will have no access to the medical records of the patients, they will instruct patients or relatives before starting the interview not to reveal the allocation (performed procedure or admission in the hospital), and they will enter the outcome data in a database which is separated from the clinical database. The final assessment of the mRS score at 90 days will be performed by the outcome committee, consisting of trained investigators blinded to the treatment allocation, based on the reports of the telephone interview. Neuroimaging results will be determined by assessors blinded to treatment allocation. All information pertaining to outcomes will be kept separately from the main study database and will remain inaccessible to the executive committee until study completion. An independent trial statistician will combine the data on treatment allocation with the clinical and outcome data to report summaries of trial progress, regular safety assessments, and interim analyses on efficacy and safety to the data safety monitoring board (DSMB).

### Intervention

Patients are assigned to either EVT plus best medical treatment or best medical treatment alone. Best medical treatment could include admission to a stroke unit or intensive care unit, secondary preventive measures, and rehabilitation. Crossover is only allowed when additional endovascular contraindications arise after randomization. Choice of an endovascular device (stent-retriever or aspiration device) will be left to the individual interventionist provided the device is CE-marked or FDA-approved for EVT (Table 2). If deemed indicated, local application of maximum dosages of alteplase (30 mg), urokinase (1.2 million IU), or abciximab (20 mg) is allowed.

In case of failed recanalization, residual intracranial stenosis or concomitant extracranial carotid pathology treatment as indicated by the intervention team, including stent placement with additional antiplatelet therapy, is allowed.

### Potential risks

The potential risks of EVT consist of intracranial and extracranial hemorrhage; procedure-related risks such as dissection, perforation, and infarctions in other vascular territories; and postprocedural events such as infections [17]. In the 5 landmark trials of 2015, the risks of hemorrhage and hemorrhagic infarction were equal for both the intervention and the control groups. Postprocedural events such as pneumonia and other infections occurred in similar frequencies in both groups, and procedure-related events were infrequent.

### Study procedures

**Fig. 4:**
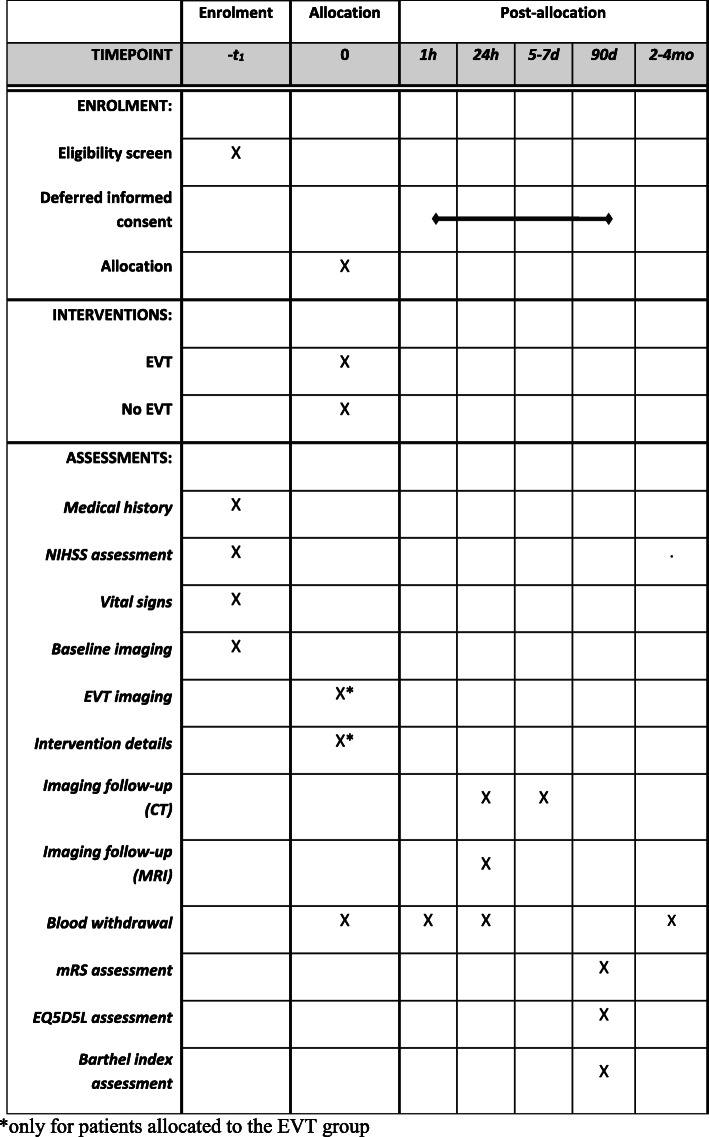
*Only for patients allocated to the EVT group

Before randomization, NIHSS score will be assessed by certified assessors. NCCT and CTA (and CTP in case of NIHSS ≥ 10) will be performed at baseline. MRA at baseline is also permitted, though collaterals must be assessed on CTA. After 24 h, further NIHSS assessment and follow-up imaging will be performed regardless of the treatment allocation. Intervention centers may choose either NCCT/CTA or MRI/MRA as a follow-up modality at 24–48 h. Participating centers should adhere to the chosen modality during the trial to prevent bias by indication. Only in case of contraindications for MRI CT imaging may be performed instead and vice versa. If follow-up imaging will be performed with MRI, DWI, FLAIR, T2*, and intracranial 3DTOF, sequences are required. If CT follow-up is chosen, NCCT will be repeated at 5–7 days or just before discharge to assess the final infarct volume. Final NIHSS score assessment will also be performed at 5–7 days or just before discharge for all included patients. Functional outcome will be assessed as mRS score, Barthel index, and EQ-5D-5L by telephone interview at 90 days after randomization by a certified research nurse. Figure 4 shows the timing of all study procedures.

Blood samples will be taken from patients when logistics at the participating centers allow this. Blood samples will be drawn at the following time points: (1) within 1 h before the EVT, (2) within 1 h after the EVT or 1 h after hospital admission in case the patient is part of the control group, and (3) at 24 h after the EVT or admission. We will also take a blood sample if the patient has a regular (none trial-related) outpatient clinic appointment (2–6 months after treatment). One tube EDTA (± 5 mL), one tube without anticoagulant (± 7 mL), and two tubes of citrated blood (2.7 mL) will be drawn, which is no more than 20 mL. Substudies may require extra blood tubes, never more than 20 mL per blood draw. When a drip is in place, which will be the case in blood drawing at moments 1, 2, and 3, this will be used. Samples will be stored at − 80 °C for later analysis of procoagulant and genetic factors that may interact with the treatment effect. In addition, this trial also makes use of “waste material”: blood aspirated during intervention with retrieved thrombi during the intervention. All biomaterials will be stored in our CONTRAST biobank for 15 years.

### Deferred consent

MR CLEAN-LATE will investigate an acute intervention in an emergency situation concerning a life-threatening disorder. For several ethical and legal reasons, the investigators ask all patients or their representatives for written consent after treatment allocation has been carried out (i.e., deferred informed consent). The patient or representative will be asked to provide consent as early as deemed appropriate and reasonable after hospital admission, ideally before upcoming study procedures after EVT and ultimately before the final outcome assessment. If a patient or his/her representative refuses to provide consent, participation in the trial will be terminated immediately. Participation in MR CLEAN-LATE is voluntary, and the patient or representative may—at any given time—withdraw informed consent without explanation. When consent by proxy has been obtained and the patient recovers, we will again ask the patient for written consent. If a patient dies before deferred consent is obtained, the representative will be informed about trial participation.

### Study outcomes

The primary outcome is the score on the modified Rankin Scale, a 7-point scale ranging from 0 (no symptoms) to 6 (death), at 90 days (± 14 days) [18]. A score of 2 points or less indicates functional independence. Secondary outcomes are as follows: (1) extended treatment in cerebral ischemia (eTICI) score on final angiography during EVT procedure [19]; (2) recanalization rate at 24 h after randomization, assessed with CTA/MRA [20]; (3) score on the NIHSS at 24 h and 5–7 days after randomization or at discharge [21]; (4) final infarct volume on MRI at 24 h or on NCCT at 5–7 days. Final infarct volume will be assessed with the use of an automated, validated algorithm [22]; (5) dichotomized mRS score of 0–1 vs 2–6 at 90 days, of 0–2 vs 3–6 at 90 days, and of 0–3 vs 4–6 at 90 days; (6) death at 90 days; and (7) score on the Barthel index and EQ-5D-5L at 90 days.

Additionally, the following safety endpoints will be assessed: (1) hemorrhages according to the ECASS classification [23]; (2) symptomatic intracranial hemorrhage (sICH) scored according to the Heidelberg criteria [24]; (3) embolization in new territory on angiography during EVT; (4) occurrence of aneurysma spurium; (5) occurrence of groin hematoma; (6) infarction in the new cerebral territory at 1 week after randomization assessed with NCCT or at 24–48 h assessed with DWI-MRI; (7) stroke progression, defined as neurological deterioration with an increase of two or more points on one NIHSS item or 4 points in total on the NIHSS and follow-up cerebral imaging compatible with the diagnosis of ischemia and no other underlying cause for neurological deterioration; and (8) all-cause mortality at 90 days.

For additional analyses in efficiency studies on EVT implementation, the following time parameters will be recorded: (1) time of onset or last seen well, (2) time of symptoms noticed, (3) time of IVT, (4) time of arrival at intervention center, (5) time of groin puncture, and (6) time of recanalization.

### (Serious) adverse event reporting

Any undesirable event occurring to a patient during the study, whether or not considered related to the experimental investigation, will be considered an adverse event. Such an event will be considered a serious adverse event (SAE) if it (1) results in death, (2) is life-threatening, (3) requires (prolonged) hospitalization, (4) results in persistent or significant disability, or (5) is a congenital anomaly or birth defect. Any other important medical event that could have resulted in any of the outcomes listed, according to appropriate medical judgment, if no medical or surgical intervention would have been carried out, will also be considered a serious adverse event. Technical complications or vascular damage at the target lesion such as perforation or dissection that does not lead to clinically detectable SAE, or neurological deterioration that is considered as consistent with the natural cause of the ischemic stroke (e.g., not caused by intracranial hemorrhage or new ischemic stroke), will be recorded but not reported immediately. The (local) investigator will report the following SAEs occurring in the study period to the sponsor without undue delay of obtaining knowledge of the events: death from any cause, symptomatic intracranial hemorrhage defined according to the Heidelberg criteria, extracranial hemorrhage, cardiac ischemia, pneumonia, allergic reactions, and new ischemic stroke in a different vascular territory. Serious adverse events that meet the aforementioned criteria will be reported to the sponsor, within 24 h after coming to notice of the (local) investigator, by making use of the appropriate forms in the eCRF, which will automatically lead to notification of the study coordinator.

### Safety registry

Due to the deferred consent procedure, study allocation and possible intervention will have taken place prior to obtaining informed consent. The procedure requires that all information on patients who did not provide consent after EVT is discarded and deleted. This may be against the interest of patients who did provide consent, and against the interest of the general public, as patients with sICH and other serious adverse events might be more likely to refuse consent for participation. Not considering these records might very well result in an underestimation of the true risk and validity of the data, and it might lead to undetected safety concerns for all consenting patients in the trial. To overcome this concern, we will register the following variables in a strictly anonymized safety registry for all patients, irrespective of whether a patient has provided written informed consent: patient’s study number, study treatment, in-hospital sICH occurrence (yes/no), and in-hospital survival status (yes/no). All other information will be completely erased from the patient’s study record in case no consent is provided. The link to the study database will be erased from the patient’s medical record.

### Data and Safety Monitoring Board

The trial will be monitored by an independent data safety monitoring board (DSMB) chaired by a neurologist, including a neuro-interventionist and an independent statistician. The DSMB will meet at least annually or after the inclusion of every 100th patient (whichever comes first) and assess the occurrence of unwanted effects by center and by procedure. Interim analyses of the major endpoints will be supplied in strict confidence in addition to any other analyses that the board may request. The executive committee will be kept unaware of the results of the interim analyses concerning efficacy and safety. The DSMB will advise the chairman of the steering committee if, in their view, the randomized comparisons in the trial have provided both (1) “proof beyond reasonable doubt” that for all, or for some specific types of patients, one particular treatment is clearly indicated or clearly contraindicated in terms of a net difference in outcome, and (2) evidence that might reasonably be expected to materially influence patient management. Appropriate criteria of proof beyond reasonable doubt cannot be prespecified precisely, but a difference of at least 3 standard deviations in an interim analysis of a major endpoint may be needed to justify halting or modifying the study prematurely. This criterion has the practical advantage that the number of interim analyses is of little importance. In case of premature termination of the study, the database will be closed after 90 days of assessment of the last enrolled patient and results will be reported.

### Sample size

We estimate that a sample size of 500 patients would provide a power of 85% with two sided alpha 0.05, using simulation with 5000 runs per assumed sample in R. For the power calculations, we assumed the distribution over the 7-point mRS in the control group which is similar to the distribution of mRS in the control group of the MR CLEAN trial [1]. We assumed a favorable effect of treatment with a common odds ratio of 1.52, which would lead to a 7% absolute increase in the proportion of patients with mRS 0–2.

### Statistical analyses

The primary effect parameter will be the common odds ratio, estimated with ordinal logistic regression, which represents the shift on the full distribution of modified Rankin Scale at 90 days. The estimate will be adjusted for the following prognostic variables: (1) age, (2) pre-stroke mRS score, (3) time from onset to randomization, (4) clinical stroke severity (NIHSS score) at baseline, (5) collateral grade, and (6) unwitnessed stroke onset. Adjusted and unadjusted estimates with corresponding 95% confidence intervals will be reported. Secondary outcomes will be analyzed using ordinal logistic regression as appropriate, with the same adjustment variables as the primary outcome.

The effect of the intervention on the modified Rankin Scale will be further analyzed in subgroups defined by (1) tertiles of age; (2) sex (m/f); (3) tertiles of systolic blood pressure at baseline; (4) tertiles of NIHSS score at baseline; (5) tertiles of time from onset to randomization, groin puncture, and revascularization; (6) diabetes mellitus; (7) atrial fibrillation; (8) extracranial carotid obstruction; (9) occlusion location (ICA, ICA-T, M1, M2/3); (10) ASPECT score (0–4, 5–7, 8–10); (11) collaterals (poor, moderate, good); (12) witnessed/unwitnessed stroke; (13) inclusion in the active treatment arm of MR ASAP; and (14) CTP characteristics. All analyses will be performed according to the intention-to-treat principle. Missing baseline characteristics will be imputed using multiple regression imputation.

A health-economic analysis will be performed to assess the cost-effectiveness of the intervention under study. The endpoint will be the cost per patient with a good functional outcome and the cost per QALY.

#### Amendments

Amendments are changes made to the research protocol after approval by the accredited METC. All amendments will be notified to the METC that gave the approval. Non-substantial amendments will not be notified to the accredited METC and the competent authority, but will be recorded and filed by the sponsor, and communicated with all study sites.

#### Data management

All MR CLEAN-LATE data are entered into a web-based trial management system that allows for edit and audit trails, by trained local research nurses. Patient records are coded by a unique study number. The local investigators will keep a list showing codes and names. Unique documents with identifying information will be stored separately from the study database in digital files, categorized by study number on a secure drive system, only accessible to the study coordinators. Data will be monitored for completeness, consistency, and validity by the study coordinators through automated data checks. Twenty-five percent of local data are carefully reviewed against source data, based on a pre-assessed risk evaluation and in accordance with the Dutch standards, by an independent monitor performing two to three visits per year during the study period (Additional file 1: Appendix 3). The database will be closed within 1 month after the last scheduled follow-up date of the last included patient.

### Study organization and funding

MR CLEAN-LATE is part of the COnsortium for New TReatments of Acute STroke (CONTRAST: Additional file 1: Appendix 1) and will be coordinated from the Maastricht University Medical Center (see Additional file 1: Appendix 2 for the MR CLEAN-LATE trial investigators). CONTRAST is supported by the Netherlands Cardiovascular Research Initiative which is supported by the Dutch Heart Foundation (CVON2015-01: CONTRAST), the Brain Foundation Netherlands (HA2015.01.06), and Health~Holland, Top Sector Life Sciences & Health (LSHM17016), Medtronic and Cerenovus. The cost-effectiveness analysis is supported by ZonMW.

### Ethical considerations

The protocol of the MR CLEAN-LATE, including the template informed consent forms, which can be found on https://www.mrclean-late.nl, has been approved by the medical ethics committee of Erasmus MC University Medical Center (MEC-2017-367) before the start of the trial. Data management, monitoring, and reporting of the trial will be performed in accordance with the ICH GCP guidelines. The study will be conducted according to the principles of the Declaration of Helsinki (October 2013), ICH-GCP, and in accordance with the Medical Research Involving Human Subjects (WMO).

## Trial status

Screening and inclusion for MR CLEAN-LATE started in January 2018 and is ongoing. In January 2021, we included 360 patients. Recruitment is expected to be completed within 4 years from the start of the trial. For up-to-date patient enrollment numbers and study sites, please visit https://www.mrclean-late.nl. The current article is based on protocol version 1.5 dating from August 2019.

### Publication policy

The study database will be closed within 1 month after the last scheduled follow-up date of the last included patient. A manuscript containing at least the study description and the answer to the primary research question will be submitted to a major clinical journal within 3 months from the closure of the database. The manuscript will be shared with the financial sponsors 1 month before submission, but they will have no influence on its contents. At the time of publication, the participants or representatives will be informed of the main results. Anonymous data can be requested from the principal investigator with a detailed description containing the aims and methods of the study for which the data are intended to be used. Data will be made available for this purpose at least 18 months after the publication of the main report.

## Discussion

MR CLEAN-LATE is a pragmatic multicenter randomized trial with PROBE design of EVT next to best medical treatment vs best medical treatment alone for large vessel occlusion acute anterior circulation stroke in patients presenting with poor, moderate, and good collaterals between 6 and 24 h from time last known well. The effect of EVT will primarily be evaluated on functional outcome and secondarily on safety aspects. To this end, the MR CLEAN-LATE will provide the basis for the pragmatic expansion of EVT to the “late” patient group. Central to this expansion will be the assessment of collateral status on CTA for EVT eligibility.

Recent publications of DAWN and DEFUSE 3 have shed light on EVT in a subpopulation of patients presenting beyond the 6-h treatment window [7, 8]. Both trials showed significant benefit for patients with large vessel anterior circulation occlusion presenting between 6–24 h and 6–16 h from time last known well, respectively. Unfortunately, there are several limitations in both trials that limit generalizability and wide implementation. Only patients with a distal carotid artery occlusion or ACM-M1 occlusion with very small infarct core sizes as selected by CT perfusion or MR diffusion assessed by use of the RAPID software were included. In DAWN, the median core size was 18 ml or less in 75% of patients. DEFUSE 3 showed core infarct sizes smaller than 26 ml in 75% of the included patient population and 75% had a penumbra larger than 80 ml. Additionally, perfusion imaging is known to suffer from wide variations in core and tissue-at-risk calculations between the various software packages used for these calculations. The use of only one software-package in DAWN and DEFUSE 3 therefore limits the generalizability in clinical practice.

It is well known that patient selection based on advanced imaging characteristics increases the chance of good functional outcome [2–5]. The MR CLEAN trial did not select on the basis of advanced imaging criteria other than proven occlusion on CTA [1]. In patients who underwent CT perfusion in MR CLEAN, CT perfusion showed an association with outcome, but not with treatment effect [25]. On the contrary, collateral status on CTA did show an interaction with treatment effect in MR CLEAN with higher collateral grades showing larger treatment effects [12].

Finally, it has been shown that of all patients with acute ischemic stroke presenting to a single comprehensive stroke center, only 1.7% of patients qualified for DAWN clinical trial enrollment with an additional 0.6 to 1% qualifying for the DEFUSE-3 trial [26]. Based on these findings, we deem it of utmost importance to perform MR CLEAN-LATE using broad inclusion criteria and widely applicable and less advanced imaging criteria.

### Expected benefit

In The Netherlands, approximately 25% of ischemic stroke patients arrive beyond the 6 h last known well time window in a hospital. Based on the results from MR CLEAN, it is expected that one in five patients will have a proximal intracranial occlusion and more than half of these will have poor to good collaterals. We therefore expect that in the 17 participating centers yearly, 1000 patients will be eligible for this study. Based on experiences in the ESCAPE trial, if one in four thereafter fulfills all other selection criteria potentially 250 patients per year will be eligible in all participating centers. We expect to find a minimum 7% absolute increase in functionally independent patients. This means that during the study period yearly, 18 patients presenting with a large vessel occlusion stroke beyond 6 h could be saved from a disabled life by EVT in The Netherlands.

### Limitations and concerns

The effectiveness and safety of EVT have already been shown in acute anterior circulation proximal vessel occlusion stroke with time from onset to groin of less than 6 h. Therefore, the risks associated with EVT are well known, and it is not expected that these risks increase with prolonged time to treatment.

It has been shown that a higher collateral score on CTA is associated with better functional outcomes after EVT. The benefit of EVT is therefore not only dependent on time but also on the presence of collaterals. Therefore, the benefit of thrombectomy outside the 6 h last known well time window in this selected patient population might be in the same range as within the 6-h time window.

In both MR CLEAN and IMS III, it has been shown that poor collateral status is associated with minimal or no treatment effect [12, 27]. Furthermore, because collaterals will decrease over time [28], it may be expected that more patients in the late time window will present with poor collaterals than in the 6-h time window. A large proportion of patients with poor collaterals in the trial may negatively affect the hypothesized treatment effect. Therefore, the total number of patients with poor collaterals will be limited to 100. The imaging core lab will evaluate baseline CTAs on a regular basis in order to monitor the number of enrolled patients with poor collateral status.

The infrastructure surrounding EVT in The Netherlands has largely been established during the MR CLEAN trial and has since then further improved, as evidenced by faster treatment times in the MR CLEAN Registry [29]. MR CLEAN-LATE can utilize the existing stroke infrastructure providing fast time metrics from presentation to treatment.

There are several CTA techniques currently in use for determining collateral status. Of these, perfusion-CTA and multiphase CTA seem to be more accurate in determining collateral grade than single-phase CTA (spCTA) [10, 11, 30, 31]. The observed difference is mostly due to an underestimation of good and excellent collateral scores by spCTA [10, 30, 31]. Absent collateral grade scores did appear similar in all groups. Therefore, the chance that patients will not be included in MR CLEAN-LATE due to a false-negative collateral score reading on spCTA will be low and acceptable. Also, the used collateral score in MR CLEAN-LATE was originally validated with spCTA. SpCTA is commonly used in the Netherlands to determine the presence of a large vessel occlusion and is therefore allowed in the current trial.

### Deferral of consent

In MR CLEAN-LATE, we use a deferred consent procedure. The primary reason for this approach is that in ischemic stroke, acute treatments are based on the “time is brain” principle, in order to reduce the loss of brain tissue as time progresses. In patients treated with EVT, each hour delay to reperfusion is associated with an increase in absolute risk of disability of 6–7% [6]. First of all, experience in MR CLEAN indicates that a proper informed consent procedure takes more than 1 h, even when a legal representative is involved. This would lead to an unacceptable delay, considering the time-dependent effect of EVT. Second, most patients with acute neurological deficits (such as impaired consciousness or aphasia) are not capable of decision making before enrollment in a trial. In the MR CLEAN Registry, 80 to 96% of the acute ischemic stroke patients eligible for EVT were in retrospect considered to lack decision-making capacity at admission, based on neurological symptoms potentially interfering with their capacity to decide about trial participation [32]. Exclusion of these patients might lead to selection bias and reduced generalizability of the trial results. Lastly, the decision-making capacity for trial participation in an emergency situation is also reduced by stress and by the complexity and volume of the provided information. Thus, the use of the deferred consent procedure is likely to increase patient enrollment and to reduce selection bias, resulting in better generalizability of the trial results. However, if a substantial number of patients or representatives object to enrollment after EVT this could actually contribute to a different kind of selection bias, particularly if this disproportionally concerns patients with adverse events and poor clinical outcome. Postponing consent seems tolerated by patients and their relatives in several clinical studies and trials [33–39]. However, a substudy of the ESCAPE trial (Endovascular Treatment for Small Core and Anterior Circulation Proximal Occlusion With Emphasis on Minimizing CT to Recanalization Times) showed that the majority of patients or their representatives disagreed with the use of deferred consent [40]. Yet, none of the patients enrolled with deferred consent in this trial withdrew consent later, and patients agreed with the conditions used to justify deferred consent procedures. A separate substudy within the CONTRAST collaboration, in the form of a survey, will be carried out to further elucidate the acceptability of the deferred consent procedure in acute stroke trials.

## Supplementary Information

**Additional file 1: Appendix 1.** Study organization. **Appendix 2.** Trial investigators. **Appendix 3.** MR CLEAN-LATE monitoring plan (Dutch).

## Data Availability

Data will be made available for replication of the study results upon reasonable request to the principal investigators, 18 months after publication of the first paper.

## Abbreviations

AIS: Acute ischemic stroke
APTT: Activated partial thromboplastin time
CONTRAST: Consortium for New Treatments of Acute Stroke
CT: Computed tomography
CTA: Computed tomography angiography
DSA: Digital subtraction angiography
DSMB: Data Safety Monitoring Board
EVT: Endovascular therapy
EQ-5D-5L: A standardized instrument developed by the EuroQol Group as a measure of health related quality of life, which comprises 5 dimensions with each 5 levels of severity
ICH: Intracranial hemorrhage
INR: International normalized ratio
IU: International standard unit
IV: Intravenous
MRI: Magnetic resonance imaging
MRA: Magnetic resonance angiography
mRS: Modified Rankin Scale
NIHSS: National Institutes of Health Stroke Scale
PROBE: Prospective Randomized Open Blinded End-point
RCT: Randomized controlled trial
(S)AE: (Serious) adverse event
sICH: Symptomatic intracranial hemorrhage
WMO: Medical Research Involving Human Subjects Act (in Dutch: Wet Medisch-wetenschappelijk Onderzoek met Mensen)
