# Supplementary material for: MR CLEAN-LATE, a multicenter randomized clinical trial of endovascular treatment of acute ischemic stroke in The Netherlands for late arrivals: study protocol for a randomized controlled trial
Source: Trials. 2021 Feb 24;22:160. doi: 10.1186/s13063-021-05092-0 (PMC7903604; doi:10.1186/s13063-021-05092-0)
Supplement: Supplementary file 1 — Additional file 1: Appendix 1. Study organization. Appendix 2. Trial investigators. Appendix 3. MR CLEAN-LATE monitoring plan (Dutch). [file 13063_2021_5092_MOESM1_ESM.zip › supplemental materialR2_ESM.pdf]

Appendix 1: Study organization

MR CLEAN-LATE is embedded in the Collaboration for New Treatments of Acute Stroke (CONTRAST) consortium, a nationwide collaboration of clinical and translational scientists. The CONTRAST consortium will perform five large RCTs in stroke patients to test novel treatment strategies; Multicenter randomized trial of acute stroke treatment in the ambulance with a nitroglycerin patch, aimed at preservation of ischemic tissue and improving outcome after stroke [MR ASAP, ISRCTN99503308]; Intravenous treatment followed by endovascular treatment versus direct endovascular treatment for acute ischemic stroke caused by a proximal intracranial occlusion [MR CLEAN-NO IV, ISRCTN80619088]; The effect of periprocedural medication: acetylsalicylic acid, unfractionated heparin, both or neither [MR CLEAN-MED, ISRCTN76741621]; Multicenter Randomized Clinical Trial of Endovascular Treatment of Acute Ischemic Stroke in The Netherlands for Late arrivals [MR CLEAN-LATE, ISRCTN19922220]; The Dutch ICH Surgery Trial - pilot study; minimally-invasive endoscopy- guided surgery for spontaneous intracerebral hemorrhage [DIST, NTR7180]. Although, MR CLEAN-NO IV, MR CLEAN-MED and MR CLEAN-LATE, which all aim to improve outcome after EVT by focusing on the optimization of EVT and the expansion of its indication, draw from the same pool of patients with acute ischemic stroke, there is no competition between the three trials (Figure 2). All studies are independent clinical trials, but investigators collaborate closely and the trials share the same data structure and format, imaging and clinical assessment procedures, and outcome, imaging and SAE assessment committees. Patients enrolled in MR CLEAN-NO IV, MR CLEAN-MED or MR CLEAN-LATE can also participate in MR ASAP, for which patients will be stratified.

The MR CLEAN-LATE is guided by several MR CLEAN-LATE-organized and CONTRAST-organized committees:

The steering committee of the trial consists of all local principal investigators (PIs) of the participating centers. Each participating center has two local PIs: a vascular neurologist and a neuro-interventionist. The steering committee will meet at least annually. Final decisions concerning protocol changes, publication and reporting will be made by the steering committee. The steering committee is chaired by the central PIs of the trial. Decisions will be made in consensus, but if unavoidable by majority vote. Day to day conduct of the trial will be managed by the trial coordinators, who will be supervised by the central PIs of the trial.
The executive committee of the trial consists of the central PIs of the trial, a representation of local PIs, including the PIs of the two other MR CLEAN II trials, and of the study coordinators. They meet regularly, discuss trial progress and prepare information for the steering committee.
The writing committee consists of the executive committee and local PIs of the five collaborating centers that have contributed the most patients to the trial in the first two years of trial execution. The task of the writing committee is to prepare the main publication which will be drafted by the study coordinators, supervised by the two central PIs. Typically, the main paper will be authored by the study coordinators, the local PIs, the committee members, the central PIs, the coordinators of the two other MR CLEAN trials, and data management group, in name of all MR CLEAN-LATE investigators. Authorship has to comply with the criteria of the International Committee of Medical Journal Editors (IMCJE at www.imcje.org).41
The other trial committees are not trial specific and will be formed in collaboration with the four CONTRAST randomized clinical trials on acute stroke: MR ASAP, MR CLEAN-NO IV, MR CLEAN-MED and MR CLEAN-LATE. These are: the imaging committee, the adverse event committee, and the outcome committee. The committees will regularly report to the steering committees of the involved trials.

The imaging committee is chaired by the CONTRAST imaging work package leaders (CM and AL) and consists of neuroradiologists from the collaborating centers. Their task is to assess and evaluate masked baseline and follow-up imaging, which is performed per protocol and stored in a central web-based database (XNAT, www.xnat.org). Assessments will be stored in research forms and entered in the clinical database, which will be accessible to investigators after approval by the Steering committee.

The adverse event committee consists of at least 3 members, including a neurologist and a neuroradiologist. Their task is to oversee and review all reported serious adverse events.

The outcome committee consists of at least 3 members, all seasoned neurologists. Their task is to evaluate all coded and masked structured reports of the outcome assessments at 90 days of patients in the trials. This way, we can ensure blind outcome assessment.

Strategies for improving adherence to the intervention protocol and other study procedures, and for achieving adequate participant enrollment include training sessions at all participating centers, regular newsletters and research meetings with all collaborators, and monthly telephone meetings with the study coordinators and central PIs of the MR ASAP, MR CLEAN-NO IV, MR CLEAN-MED, and MR CLEAN-LATE.

Appendix 2: Trial investigators

The MR CLEAN-LATE investigators

Principal investigators

Robert J. van Oostenbrugge (MD, PhD)[1], Wim H. van Zwam (MD, PhD)[1]

Study Coordinators

F.A.V. (Anne) Pirson (MD)[1], Wouter H. Hinsenveld (MD)[1], Susan G.H. Olthuis (MD)[1]

Robert-Jan B. Goldhoorn (MD,PhD)[1]

Local principal investigators

Robert J. van Oostenbrugge (MD, PhD)[1], Wim H. van Zwam (MD, PhD)[1], Julie Staals (MD, PhD)[1], Diederik W.J. Dippel (MD, PhD)[2], Aad van der Lugt (MD, PhD)[2], Adriaan van Es, (MD, PhD)[2], Bob Roozenbeek (MD, PhD)[2], Yvo B.W.E. Roos (MD, PhD)[3], Charles B.L.M. Majoie (MD, PhD)[3], Jonathan M. Coutinho (MD, PhD)[3], Bart Emmer (MD, PhD)[3], Bart van der Worp (MD, PhD)[4], Rob T. Lo (MD, PhD)[4], Marianne A.A. van Walderveen (MD, PhD)[5], Marieke J. Wermer (MD, PhD)[5], Ewoud I. van Dijk (MD, PhD)[6], Sjoerd Jenniskens (MD, PhD)[6], Maarten Uyttenboogaart (MD, PhD)[7], Reinoud P.H. Bokkers (MD, PhD)[7], Koos Keizer (MD, PhD)[8], Rob Gons (MD, PhD)[8], Lonneke Yo (MD, PhD)[8], Heleen den Hertog (MD, PhD)[9], Boudewijn van Hasselt (MD)[9], Wouter J. Schonewille (MD, PhD)[10], Jan-Albert Vos (MD, PhD)[10], Julia H. van Tuijl (MD)[11], Issam Boukrab (MD)[11], Jeannette Hofmeijer (MD, PhD)[12], Jasper Martens (MD)[12], Ido R. van den Wijgaard (MD, PhD)[13], Jelis Boiten (MD, PhD)[13], Paul J.A.M. Brouwers (MD, PhD)[14], Emiel J.C. Sturm (MD)[14], Karlijn F. de Laat (MD, PhD)[15], Lucas C. van Dijk (MD, PhD)[15], Michel J.M. Remmers (MD)[16], Thijs E.A.M. de Jong (MD)[16], Anouk Rozeman (MD)[17], Otto E.H. Elgersma (MD, PhD)[17]

Trial collaborators:

Executive committee

Robert van Oostenbrugge (MD, PhD)[1], Wim van Zwam (MD, PhD)[1], Marianne van Walderveen (MD, PhD)[5], Geert Lycklama à Nijeholt (MD, PhD)[13], Wouter Schonewille (MD, PhD)[10], Maarten Uyttenboogaart (MD, PhD)[7], Charles Majoie (MD, PhD)[3]

Data monitoring committee

Heinrich Mattle (MD,PhD)[18] – *Chair*, professor of Neurology

Jens Fiehler (MD, PhD)[19], professor of Interventional Neuroradiology

Sander van Kuijk (PhD)[1], statistician, Department of Epidemiology

Independent trial statistician

Daan Nieboer (MSc)[2]

Contrast Clinical trial collaborators:

Research leaders

Diederik Dippel (MD, PhD)[2], Charles Majoie (MD, PhD)[3]

Consortium coordinator:

Rick van Nuland (PhD)[20]

Imaging assessment committee

Charles Majoie (MD, PhD)[3] – *Chair*, Aad van der Lugt (MD, PhD)[2] – *Chair*, Wim van Zwam (MD, PhD)[1], Alida Postma (MD, PhD)[1] , René van den Berg, (MD, PhD)[3], Ludo Beenen (MD)[3], Bart Emmer (MD, PhD)[3], Adriaan van Es, (MD, PhD)[2], Pieter-Jan van Doormaal (MD)[2], Geert Lycklama (MD, PhD)[13], Ido van den Wijngaard (MD, PhD)[5], Albert Yoo (MD, PhD)[21], Lonneke Yo (MD, PhD)[8], Jasper Martens (MD)[12], Sebastiaan Hammer (MD, PhD)[15], Stefan Roosendaal (MD, PhD)[3], Anton Meijer (MD, PhD)[6], Menno Krietemeijer (MD)[8], Reinoud Bokkers (MD, PhD)[7], Anouk van der Hoorn (MD, PhD)[7], Dick Gerrits (MD)[14]

Adverse event committee

Robert van Oostenbrugge (MD, PhD)[1] – *Chair*, Bart Emmer (MD, PhD)[3], Jonathan Coutinho (MD, PhD)[3], Ben Jansen (MD)[11]

Outcome assessment committee

Yvo Roos (MD, PhD)[3] – *Chair,* Sanne Manschot (MD, PhD)[13], Diederik Dippel (MD, PhD)[2], Henk Kerkhof (MD, PhD)[17], Ido van den Wijngaard (MD, PhD)[5], Jonathan Coutinho (MD, PhD)[3], Peter Koudstaal (MD, PhD)[2], Koos Keizer (MD, PhD)[8], Jelis Boiten (MD, PhD)[13]

Data management group

Hester Lingsma (PhD)[2], Diederik Dippel (MD, PhD)[2], Vicky Chalos (MD)[2], Olvert Berkhemer (MD, PhD)[2]

Imaging data management

Aad van der Lugt (MD, PhD)[2], Charles Majoie (MD, PhD)[3], Adriaan Versteeg[2], Lennard Wolff (MD)[2], Jiahang Su (MSc)[2],

Biomaterials and translational group

Hugo ten Cate (MD, PhD)[1], Moniek de Maat (PhD)[2], Samantha Donse-Donkel (MD)[2], Heleen van Beusekom (PhD)[2], Aladdin Taha (MD)[2]

Local collaborators

Vicky Chalos (MD)[2], Kilian Treurniet (MD)[3], Sophie van den Berg (MD)[3], Natalie LeCouffe (MD)[3], Rob van de Graaf (MD)[2], Robert-Jan Goldhoorn (MD)[1], Aladdin Taha (MD)[2], Samantha Donkel (MD)[2], Wouter Hinsenveld (MD)[1], Anne Pirson (MD)[1], Lotte Sondag (MD)[6], Susan Olthuis (MD)[1], Florentina Pinckaers (MD)[1], Manon Kappelhof (MD)[3], Rik Reinink (MD)[4], Simone Uniken Venema (MD)[4],Manon Tolhuisen (MD)[3], Laura van Meenen (MD)[3], Lennard Wolff (MD)[2], Sabine Collette (MD)[7], Wouter van der Steen (MD)[2]

Research nurses

Sabrina Verheesen[1], Martin Sterrenberg[2],Naziha El Ghannouti[2], Rita Sprengers[3], Wilma Pellikaan[10], Yvonne Drabbe[15], Joke de Meris[13], Michelle Simons[12], Hester Bongenaar[8], Anja

van Loon[16], Karin Kraus[16], Eva Ponjee[9], Rieke Eilander[9], Suze Kooij[17], Marieke de Jong[7], Friedus van der Minne[7], Esther Santegoets[11]

Study monitors

Leontien Heiligers[2], Yvonne Martens[2]

Independent expert

Bart Jacobs (MD, PhD)[2]

Affiliations

1 Cardiovascular Research Institute Maastricht (CARIM), Maastricht University Medical Center, Maastricht, The Netherlands;

2 Erasmus MC University Medical Center, Rotterdam, the Netherlands;

3 Amsterdam Medical Center, location AMC, University of Amsterdam, Amsterdam the Netherlands;

4 University Medical Center Utrecht, Brain Center Rudolf Magnus, Utrecht, the Netherlands;

5 Leiden University Medical Center, the Netherlands;

6 Radboud University Medical Center, Nijmegen, the Netherlands;

7 University Medical Center Groningen, the Netherlands;

8 Catharina Hospital, Eindhoven, the Netherlands;

9 Isala Klinieken, Zwolle, the Netherlands;

10 St. Antonius Hospital, Nieuwegein, the Netherlands;

11 Elisabeth-TweeSteden Hospital, Tilburg, the Netherlands;

12 Rijnstate Hospital, Arnhem, the Netherlands;

13 Haaglanden Medical Center, the Hague, the Netherlands;

14 Medisch Spectrum Twente, Enschede, the Netherlands;

15 HagaZiekenhuis, the Hague, the Netherlands;

16 Amphia Hospital, Breda, the Netherlands;

17 Albert Schweitzer Hospital, Dordrecht, the Netherlands;

18 University Clinic for Neurology, Inselspital Bern, Switzerland;

19 University of Hamburg, Germany;

20 Lygature, Utrecht, the Netherlands;

21 Texas Stroke Institute, Plano, Texas, United States of America

Appendix 3 MR CLEAN-LATE monitoring plan (Dutch)

See PDF
